# Modulation of Murine Olivary Connexin 36 Gap Junctions by PKA and CaMKII

**DOI:** 10.3389/fncel.2017.00397

**Published:** 2017-12-14

**Authors:** Paolo Bazzigaluppi, Sheena C. Isenia, Elize D. Haasdijk, Ype Elgersma, Chris I. De Zeeuw, Ruben S. van der Giessen, Marcel T. G. de Jeu

**Affiliations:** Department of Neuroscience, Erasmus Medical Center, Rotterdam, Netherlands

**Keywords:** inferior olive, electrical synapse, PKA, αCaMKII, β-CaMKII, tracer-coupling

## Abstract

The inferior olive (IO) is a nucleus located in the brainstem and it is part of the olivo-cerebellar loop. This circuit plays a fundamental role in generation and acquisition of coherent motor patterns and it relies on synchronous activation of groups of Purkinje cells (PC) in the cerebellar cortex. IO neurons integrate their intrinsic oscillatory activity with excitatory inputs coming from the somatosensory system and inhibitory feedback coming from the cerebellar nuclei. Alongside these chemical synaptic inputs, IO neurons are coupled to one another via connexin 36 (Cx36) containing gap junctions (GJs) that create a functional syncytium between neurons. Communication between olivary neurons is regulated by these GJs and their correct functioning contributes to coherent oscillations in the IO and proper motor learning. Here, we explore the cellular pathways that can regulate the coupling between olivary neurons. We combined *in vitro* electrophysiology and immunohistochemistry (IHC) on mouse acute brain slices to unravel the pathways that regulate olivary coupling. We found that enhancing the activity of the protein kinase A (PKA) pathway and blocking the Ca^2+^/calmodulin-dependent protein kinase II (CaMKII) pathway can both down-regulate the size of the coupled network. However, these two kinases follow different mechanisms of action. Our results suggest that activation of the PKA pathway reduces the opening probability of the Cx36 GJs, whereas inhibition of the CaMKII pathway reduces the number of Cx36 GJs. The low densities of Cx36 proteins and electrical synapses in βCaMKII knock-out mice point towards an essential role for this protein kinase in regulating the density of GJs in the IO. Thus, the level of olivary coupling is a dynamic process and regulated by a variety of enzymes modulating GJs expression, docking and activity.

## Introduction

The olivocerebellar system is essential for coordination of movements and is tuned by synchronous firing between bands of Purkinje cells (PC) in the cerebellar cortex (Welsh, 2002; Shin et al., 2007). Timing of complex spike firing of cerebellar PCs depends on the synchronizing input coming from the inferior olive (IO). The generation of action potentials (AP) of IO neurons depends on subthreshold oscillations of the membrane potential (mV) and their synchronization is promoted by Connexin 36 (Cx36) containing gap junctions (GJs; i.e., electrical synapses). Blocking electrical synapses in the IO has been shown to decrease the synchronicity of complex spike firing in the PCs (Blenkinsop and Lang, 2006). Moreover, disruption of olivary coupling affects the firing behavior of olivary neurons, increasing the occurrence of doublets and thereby corrupting the ability to respond with acute motor reflexes or acquire new motor behaviors (Van Der Giessen et al., 2008; Bazzigaluppi et al., 2012; De Gruijl et al., 2014). All these results suggest that the coupling between IO neurons is fundamental for correct functioning of the olivocerebellar system and consequently for proper execution of motor commands (Llinás, 2011). So far, the role of electrotonic coupling in the IO has been investigated via genetic (Placantonakis et al., 2002; Van Der Giessen et al., 2008) or direct pharmacological (Long et al., 2002; Leznik and Llinás, 2005) manipulations aiming to knock-out or block GJs. The final goal of these studies was to explore the physiological consequences of the lack of electrotonic coupling between olivary neurons. However, these studies did not identify the cellular mediators that can modulate electrotonic coupling between IO neurons at a more structural level. In this study (Bazzigaluppi, 2013), we focus on two pathways that might be able to control electrotonic coupling between olivary neurons on a more long-term basis. In the retina, Cx36 is known to be phosphorylated by protein kinase A (PKA) and this mechanism constitutes a way to uncouple amacrine and bipolar cells (Urschel et al., 2006). Likewise, in the IO Ca^2+^/calmodulin-dependent protein kinase II (CaMKII), which is able to phosphorylate Cx36 (Alev et al., 2008; Siu et al., 2016), is required for NMDAR-dependent strengthening of electrical coupling between olivary neurons (Turecek et al., 2014). Taken together, these lines of evidence led us to hypothesize a role for both PKA and CaMKII in regulating the coupling between olivary neurons. In the present study, we used the tracer transfer method (Abbaci et al., 2008) in combination with pharmacological agents that affect PKA and CaMKII pathways to investigate their role in structurally controlling electrotonic coupling between IO neurons. Furthermore, immunohistochemical as well as ultrastructural analysis of GJs was performed on olivary slices of αCaMKII and βCaMKII knock-out mice to investigate the importance of these enzymes for the insertion of Cx36 proteins and the formation of electrical synapses. Our results demonstrate that activating the PKA pathway or inhibiting the CaMKII pathway down-regulates coupling between olivary neurons. Activating the PKA pathway might reduce the opening probability of the Cx36 GJs, whereas inhibition of the CaMKII pathway might reduce the number of Cx36 GJs. The latter was also confirmed in βCaMKII knock-out mice, but not αCaMKII knock-out mice, suggesting involvement of the actin cytoskeleton in the olivary glomerulus.

## Materials and Methods

C57BL/6 male mice were imported from Harlan. CaMKII mutant mice were generated and bred at the Erasmus Medical Center (Elgersma et al., 2002; van Woerden et al., 2009). Animals were housed at Erasmus Medical Center in a 12 h light-dark regime. Food and water were provided *ad libitum*. All animal procedures were approved by the Dutch Ethical Committee (DEC) at Erasmus Medical Center (ID: 115-08-16).

### *In Vitro* Tracer Transfer Method

Coronal slices of the brainstem of mice (age: 3- to 6-weeks) were cut with a vibroslicer (Leica VT1000) and were transferred to a storage chamber to be incubated in ACSF containing (in mM): 124 NaCl, 5 KCl, 1.25 Na_2_HPO_4_, 2.5 MgSO_4_, 2 CaCl_2_, 26 NaHCO_3_ and 20 D-glucose, bubbled with 95% O_2_ and 5% CO_2_ (all chemicals were purchased from Sigma-Aldrich). After an incubation period of at least 1 h a slice was placed in the recording chamber through which ACSF (34–35°C) flowed at 6–7 ml/min. Whole-cell patch-clamp recordings were performed using an EPC-10 amplifier (HEKA Electronics, Germany). The patch pipettes were filled with intracellular solution containing (in mM): 120 K-gluconate, 9 KCl, 10 KOH, 3.48 MgCl_2_, 4 NaCl, 10 HEPES, 4 Na_2_ATP, 0.4 Na_3_GTP, and 17.5 sucrose. Neurobiotin tracer (final concentration: 1%, Vector Labs) was added to the intracellular solution. The pH of the intracellular solution was adjusted to 7.25. Input resistance (Ri) was measured by injection of hyperpolarizing test currents (200 pA; 100 ms) and was calculated from the voltage transient toward the end of current injection. Neurons were excluded if the input resistance varied by >15%. Data was analyzed in Clampfit 9.2 (Axon Instruments, Foster City, CA, USA). The neurons were held in whole-cell configuration for 10 min and then the electrode was gently retracted allowing the cell membrane to form a gigaseal again. The electrode was removed leaving the neuron in place and the slice was then moved to the recovery chamber in order to allow the tracer to diffuse for 260 min. At the end of the diffusion period the slice was immersed in a paraformaldehyde (PFA; 4%) solution for 1 h and then transferred to a phosphate buffer (PB 0.1 M) before being processed for immuno-histochemistry.

The tracer transfer method was executed under four different conditions. In control condition, brain slices were maintained before, during and after the tracer injection exclusively in normal ACSF. In all the other conditions, brain slices were pre-incubated (at least 1 h before the tracer injection), injected and recovered in ACSF containing either Forskolin (10 μM), or KN93 (10 μM), or H-89 (1 μM). A total of 47 IO neurons were successfully held in whole cell configuration for 10 min and filled with Neurobiotin. Resting mV, input resistance and membrane capacitance were measured as described in our previous works (Khosrovani et al., 2007). AP were evoked in current clamp with five 300 ms steps of increasing amplitude (100 pA) every 5 s. Maximal firing frequency was calculated as the inverse of the inter-spike interval between the first and the second AP evoked with the injection of 500 pA. Current step protocol entails the injection of depolarizing currents, which are known to down-modulate the GJs (Haas et al., 2011). To limit the influence of current injections, we applied the above mentioned protocol only four times to all the recorded neurons in all conditions.

### Immunohistochemistry

Tracer transfer of Neurobiotin was visualized with immunohistochemistry (IHC) on brainstem slices in which electrophysiological experiments were successfully performed. Additionally, Cx36 GJs were visualized in these slices using IHC. The procedure was performed at room temperature and pH of all solutions was 7.4. Slices were washed twice with PB (0.1 M) and the tissue was incubated in normal horse serum (10%) and Triton-X100 (0.5%) for 2 h. In order to identify Cx36 GJs, free floating slices were labeled with goat polyclonal anti-Cx36 antibody (Santa Cruz Biotechnology, Santa Cruz, CA, USA; 1 ug/ml) for 96 h at 4°C. After incubation with primary antibody, IO slices were first rinsed four times for 10 min in PB (0.1 M) then incubated for 4 h with donkey anti-goat Cy3 conjugated antibody (Jackson Immunoresearch, West Grove, PA, USA; 1:200), and Alexa Fluor 488 conjugated Streptavidin (Invitrogen, 1:200); the latter incubation procedure is to attach a fluorescent signal to the Neurobiotin tracer. Incubation with secondary antibodies was followed by four wash steps of 10 min with PB (0.1 M). Finally, slices were mounted on coverslips, embedded in Vectashield mounting medium and stored in dark at 4°C until further use.

### Confocal Imaging and Tracer Transfer Analysis

**Figure 1:**
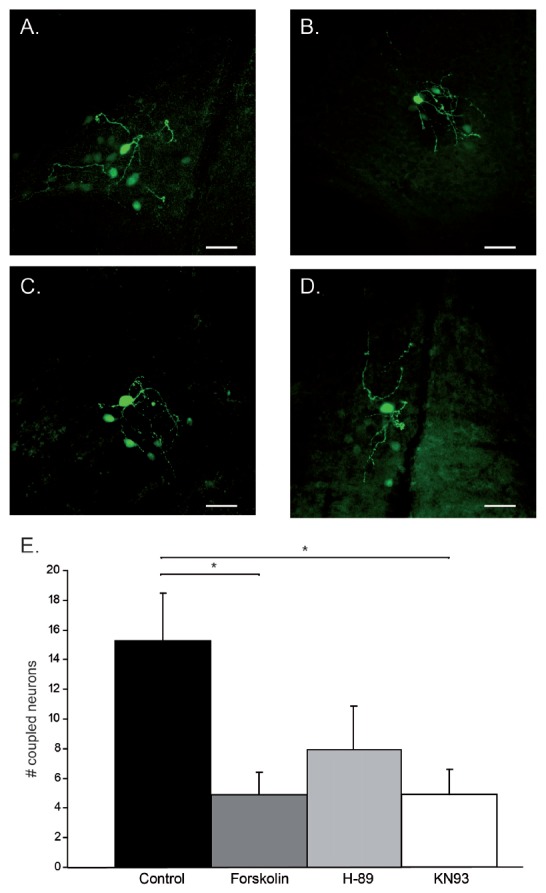
Modulation of tracer-coupling by pharmacological intervention. **(A)** Control condition: besides the primary stain cell, 13 tracer-coupled neurons can be observed. **(B)** Forskolin condition: besides the primary stain cell, four tracer-coupled neurons can be observed. **(C)** H-89 condition: besides the primary stain cell, four tracer-coupled neurons can be observed. **(D)** KN93 condition: besides the primary stain cell, eight tracer-coupled neurons can be observed. In all cases, scale bar is 25 μm. **(E)** Histogram represents the average (+SEM) number of tracer-coupled neurons in each of the four conditions. Asterisks indicate a statistical significance difference (*post hoc* Tukey HSD test *p* < 0.05, *n* = 12 in Control, Forskolin and H-89, *n* = 11 in KN93).

To determine the size of the tracer-coupled neuronal network and the distribution of Cx36 GJs, confocal images were obtained using a Zeiss LSM700 confocal laser scanning microscope operated by standard Zen 2009 software. First, a z-stack that included all visible tracer-coupled IO neurons surrounding the primary filled neuron was obtained with a 20× objective (see Figure 1D); planes were separated by optimal z-scaling automatically determined by the operation software. Confocal images were opened in Zen 2009 Light edition and tracer-coupled neurons were manually quantified by two researchers, who had no knowledge of the pharmacological treatment of the slices.

### Cx36 Puncta Quantification

**Figure 2:**
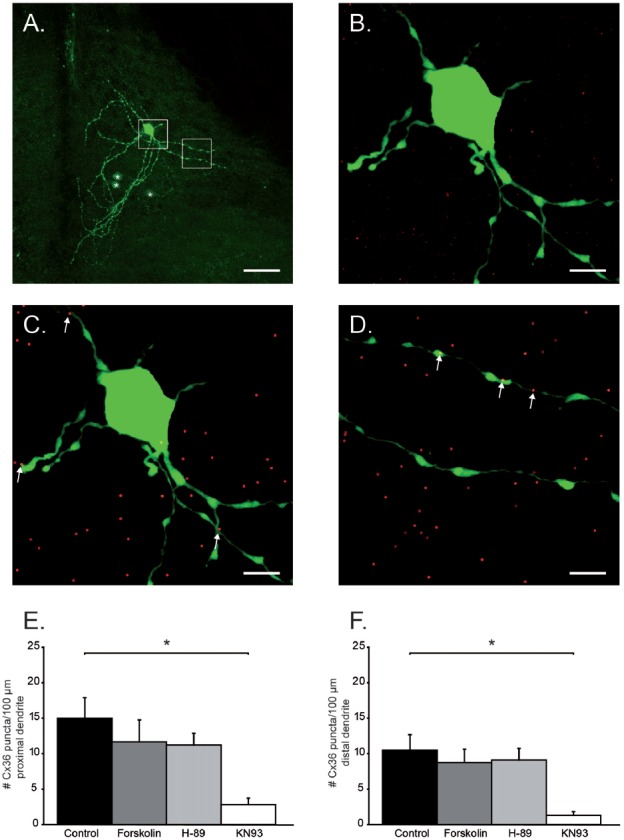
Quantification of Cx36 puncta on proximal and distal dendrites. **(A)** Example of a primary neuron and its dendritic tree. Asterisks indicate coupled neurons. White boxes are the areas chosen for Cx36 quantification and are enlarged in **(B–D)**. Scale bar is 25 μm. **(B)** Histochemical image of soma and proximal dendritic branches of olivary neuron. Green channel: neurobiotin-staining; Red channel: Cx36 GJ staining. **(C)** Same as in **(B)** after center of mass-based Cx36-recognition algorithm. Cx36 positive puncta (dots) that were located on the proximal dendrites are indicated by the white arrows. **(D)** Distal dendritic branches after center of mass-based Cx36-recognition algorithm. Cx36 positive puncta (dots) that were located on the distal dendrites are indicated by the arrows. In **(B**–**D)** scale bar is 6.25 μm. **(E)** Histogram represents the average (+SEM) number of Cx36 puncta/100 μm proximal dendrite (asterisk indicates statistical significance difference, *post hoc* Tukey HSD test *p* < 0.05). **(F)** Histogram represents the average (+SEM) number of Cx36 puncta/100 μm distal dendrite (asterisk indicates statistical significance difference, *post hoc* Tukey HSD test *p* < 0.05).

To study the localization of Cx36 puncta on dendrites of the primary Neurobiotin-injected IO neurons, two channel images were made using a 63× objective and 2× digital zoom; one image in which the Neurobiotin-filled dendrites were excited at 488 nm and one image in which the Cx36 GJs were excited at 555 nm. For each primary cell, three z-stack scans of proximal and three of distal dendritic trees were made. Dendritic branches up to 50 μm from the soma were considered proximal, while further than 70 μm were considered distal. Three proximal and three distal segments lying in the same focal plane were analyzed in every neuron. Cx36 puncta were quantified using Fiji software (ImageJ: image processing software), the two channel image was first separated in two images of one channel (an image of the dendritic tree and an image of the Cx36 GJ puncta). The length of dendritic tree was measured in three dimensions with the simple neurite tracer function preprogrammed in Fiji software. Cx36 puncta were detected using the 3D object counter function. Center of mass of the puncta was determined using threshold setting of 50 and size between 10 and 500 voxels (corresponding with a puncta size range of ~0.3–1.2 μm). Puncta corresponding to these criteria were selected and considered part of Cx36 GJs. Subsequently, images with the dendritic trees and the selected Cx36 puncta represented by center of masses objects were merged into a one channel image (Figures 2B–D). Center of masses that co-localized with measured dendritic tree were then manually counted with the use of the count cells function of Fiji software. Two researchers, who had no knowledge of the pharmacological treatment of the slice, counted the number Cx36 puncta for each dendritic tree. An average of the amount of Cx36 puncta per 100 μm dendrite was calculated for both proximal and distal dendritic trees of each primary tracer-filled IO neuron.

To determine the density of Cx36 puncta in slices of αCaMKII−/−, βCaMKII−/− mutants and their wild-type (WT) littermates, Cx36 protein were labeled with goat polyclonal anti-Cx36 antibody (Santa Cruz Biotechnology, Santa Cruz, CA, USA) as describe above. Volumetric samples (10.6 10^3^ μm^3^) were taken from these slices by making 16 z-stack scans (excited at 555 nm; 63× objective; 2× digital zoom) of 51.4 by 51.4 μm that were separated by 0.25 μm. In these samples, all Cx36 puncta were detected and quantified by using Fiji software and the procedure described above. From each mouse three samples were taken from the IO and one background sample was taken just outside the IO. The density of puncta from the background sample was subtracted from the IO samples.

### Ultrastructural Analysis of Electrical Synapses

**Figure 3:**
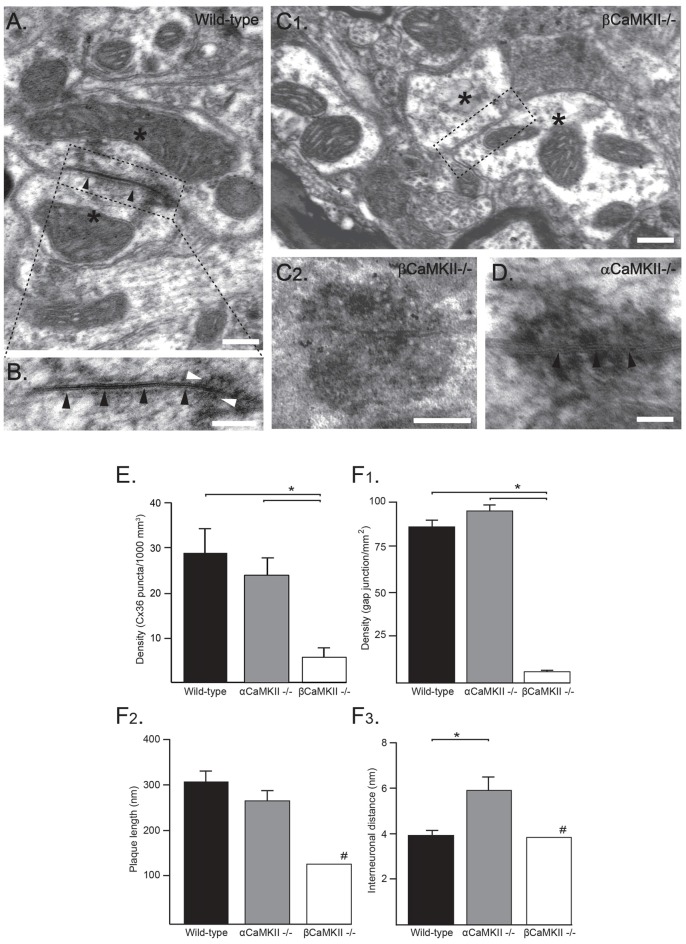
Ultrastructural analysis of electrical synapses in the inferior olive (IO) of wild-type (WT) and CaMKII mutant mice. **(A)** Ultrastructural characteristics of an olivary glomerulus in the IO of a WT mouse. The Gap junctions (GJs) between olivary dendritic spines (asterisks) showed electron-dense deposits (in **B** white arrowheads) in the cytoplasm at both sides of the membrane and attachment plaques surrounding the plaque (black arrowheads) with Cx36-proteins. **(B)** Higher magnification of the inset depicted in **(A)**. Scale bars: **(A)** 100 nm; **(B)** 200 nm. **(C1)** Ultrastructural morphology of a olivary glomerulus of homozygous βCaMKII-deficient mice. In nearly all glomeruli, no GJ structures were observed, but instead showed two dendritic spines (see asterisks) in the center surrounded by axonal terminals without a neuronal GJ (dashed box in **C1**, Scale bar: 200 nm). **(C2)** In one olivary glomerulus an adhesion-plaque structure was observed without a narrow interneuronal space as in normal GJs (Scale bar: 200 nm). **(D)** In αCaMKII- deficient mice, GJs exhibited a normal distance between attachment plaques (black arrowheads), however their average interneuronal space was significantly wider (Scale bar: 100 nm). **(E)** Histograms showing density of Cx36 puncta in βCaMKII−/−, αCaMKII−/− and WT mice determined by using immunohistochemistry (IHC). Asterisk indicates statistical significance difference (*post hoc* Tukey HSD test *p* < 0.01). **(F)** Histograms showing morphometrics of GJs in βCaMKII−/−, αCaMKII−/− and WT mice (#: in βCaMKII-deficient mice only one GJ was observed therefore no error bar is depicted). **(F1)** The average density of GJs in βCaMKII−/− mice was significantly smaller than that of olivary subnuclei in WT and αCaMKII−/− mice. **(F2)** The length of the gap-junction plaque in βCaMKII−/− mice was significantly shorter than that of WT mice, whereas in homozygous αCaMKII-deficient mice it was comparable to WT mice. **(F3)** The interneuronal distance did not differ among WT and βCaMKII−/− mice, however the average interneuronal distance of αCaMKII-deficient mice was significantly larger. Asterisk indicates statistical significance difference (*post hoc* Tukey HSD test *p* < 0.05).

Adult αCamkII−/−, βCamKII−/− mutant mice and WT littermates were anesthetized with an overdose of Nembutal and transcardially perfused with 4% paraformaldehyde and 1% glutaraldehyde in 0.12 M cacodylate buffer; the brainstem containing the IO was processed for electron microscopy. The sections of 100 μm were cut on a vibratome, osmicated in OsO_4_, stained en bloc in tannic acid and uranyl acetate, dehydrated in dimethoxypropane, and embedded in Araldite. Subsequently, various olivary subnuclei were identified in semi-thin sections, pyramids were made, and ultrathin sections were cut accordingly on a Reichert ultratome, counterstained with uranyl acetate and lead citrate, and examined with the use of a Philips CM-100 electron microscope. The sections of the various tissue blocks were screened per surface area, and GJs were identified and quantified by a researcher who had no knowledge about the origin of the tissue. GJs were identified by their morphology; a membrane plaque with a small interneuronal pace (Figures 3A,B, black arrowheads) with electron-dense deposits on both sides (Figure 3B, white arrowheads).

### Statistics

Data from the αCamkII WT littermates and βCamKII WT littermates were merged and is indicated as the WT littermate group. The results are expressed as average ± SEM (unless noted otherwise). Statistical significance has been assessed by using one-way ANOVA, *post hoc* Tukey’s HSD and Bonferroni tests and Dunnett’s test (SPSS software). Given the consistency between the two approaches, only Tukey’s HSD test’s results have been reported.

## Results

In the present work, we explore two pathways controlling the coupling between olivary neurons. The extension of the coupled network and the mean number of Cx36 GJs on olivary neurons’ dendrites were determined in slices that were incubated under four different conditions. Control slices (untreated) were compared to slices treated either with Forskolin (activator of PKA), or H-89 (inhibitor of PKA), or KN93 (inhibitor of CaMKII). In all four experimental conditions, basic membrane properties of olivary neurons were measured: input resistance (*R*_in_), resting mV (*V*_m_), membrane capacitance (*C*_m_) and maximal firing frequency (Table 1). Although we expected that the drugs would have an effect on *R*_in_ and *C*_m_, none of the membrane properties was significantly affected by the presence of any of these drugs (one-way ANOVA and Tukey’s HSD test, *p* values shown in Table 1). For this reason, we concluded that drug effects observed in the number of coupled neurons are not well represented by alteration in membrane properties of IO neurons (Prinz and Fromherz, 2003).

**Table 1:** Basic membrane properties are compared between control group and the Forskoline, H-89 and KN93 groups.

	Control (*n* = 12)	Forskoline (*n* = 12)	H-89 (*n* = 12)	KN93 (*n* = 11)	ANOVA *F*_(3,43)_ (*p*-value)
Maximal firing frequency (Hz)	18.6 ± 4.4	16.2 ± 4.3	12.9 ± 4.7	12.4 ± 2.2	0.43 (0.73)
Membrane capacitance (pF)	161.6 ± 58.4	104.0 ± 7.9	110.5 ± 22.3	181.4 ± 53.0	0.80 (0.50)
Input resistance (MΩ)	98.9 ± 14.8	123.0 ± 23.5	109.2 ± 24.6	172.3 ± 41.4	1.41 (0.25)
Resting membrane potential (mV)	−59.7 ± 1.7	−63.0 ± 5.3	−64.1 ± 8.4	−60.2 ± 1.7	1.19 (0.33)

*The last column reports the p-values for the one-way ANOVA. None of the electrophysiological parameters showed a significant interaction with the treatment (p-value between brackets)*.

### Modulation of Tracer-Coupled Networks

Generally, the tracer-coupled network appears as a group of neuronal somata stained with different intensity (Figure 1A). To explore the modulation of Cx36 GJ in neuronal network formation in the IO, the size of the tracer-coupled network was studied under the above mentioned four different conditions. Our data revealed a significant interaction between the treatment and the size of the tracer-coupled network (one-way ANOVA, *F*_(3,43)_ = 4.2, *p* < 0.05). In untreated slices, Neurobiotin distribution showed that the primary stained neuron is on average tracer-coupled to 15.3 ± 3.2 neurons (*n* = 12; Figures 1A,E). In the group of slices that were treated with Forskolin, a significant reduction in the number of coupled neurons was shown with an average of 4.9 ± 1.5 tracer-coupled neurons (*n* = 12; Figures 1B,E; Tukey’s HSD *post hoc* test, *p* < 0.05). This result indicates that an increased PKA activity down-regulates the coupling between IO neurons. Alternatively, the possibility that the inhibition of PKA activity increases the number of coupled neurons was explored by incubating slices with H-89. In those slices, the number of tracer-coupled neurons was not significantly different from the number of tracer-coupled neurons observed in the untreated slices (*n* = 12; Figures 1D,E; Tukey’s HSD *post hoc* test, *p* = 0.15). On average the primary cell was tracer-coupled to 7.9 ± 2.9 neurons in H-89 treated slices. Finally, CaMKII as Cx36 GJ modulator was investigated by blocking its activity with KN93 (Vest et al., 2007). This block of CaMKII activity significantly reduced the number of tracer-coupled neurons when compared to the number of tracer-coupled neurons that were measured in untreated slices (*n* = 11; Figures 1C,E; Tukey’s HSD *post hoc* test, *p* < 0.05). On average the primary cell was tracer-coupled to 4.9 ± 1.7 neurons in KN93 treated slices. Overall, our results show that both the PKA and CaMKII pathway can modulate the size of the coupled network.

### Modulation of Dendritic Cx36 Gap Junctions

The activation of PKA and the inhibition of CaMKII both caused a reduction in number of tracer-coupled neurons. This reduction can either be due to a reduced opening probability of GJ channels or by a reduced availability/presence of GJ channels at electrical synaptic sites. In order to elucidate the mechanism that is responsible for this reduction of tracer-coupling, Cx36 puncta on the proximal and distal dendrites of primary stained IO neurons were visualized and quantified. The primary neuron of coupled network is the whole-cell recorded neuron that is directly loaded with Neurobiotin tracer, and for this reason it always presents clear visible dendritic arborizations (Figure 2A). The length of proximal and distal dendritic branches of each primary neuron were measured (Figure 2A white boxes, Figure 2B) and the number of Cx36 puncta expressed on those dendritic fragments were counted (Figures 2C,D, see “Materials and Methods” section). For both the proximal and distal dendrites, a significant interaction was found between the treatment and the number of Cx36 puncta (proximal: one-way ANOVA, *F*_(3,35)_ = 3.37, *p* < 0.05; distal: one-way ANOVA, *F*_(3,35)_ = 3.48, *p* < 0.05). On proximal dendrites, the number of Cx36 puncta is 14.9 ± 2.9/100 μm (*n* = 15) in untreated slices and it is slightly but not significantly reduced in the slices treated either with Forskolin or H-89 (11.7 ± 3.1/100 μm, *n* = 7, *p* = 0.83 and 11.2 ± 1.7/100 μm, *n* = 10, *p* = 0.71 respectively, Tukey HSD *post hoc* test). However, when slices were incubated with KN93, the number of Cx36 GJ puncta was significantly reduced compared to the untreated condition (Tukey HSD *post hoc* test, *p* < 0.01). On average the proximal dendrites expressed 2.8 ± 0.9 Cx36 puncta/100 μm in KN93 treated slices (*n* = 7, Figure 2E). Comparable results were obtained when counting Cx36 puncta on distal dendrites, although absolute values are slightly lower for the counting of distal dendrites; the number of Cx36 puncta is 10.5 ± 2.2/100 μm dendrite in untreated slices (*n* = 15), 8.8 ± 1.9/100 μm dendrite when treated with Forskolin (*n* = 7) and 9.1 ± 1.7/100 μm dendrite when treated with H-89 (*n* = 10). The number of Cx36 GJs puncta/100 μm dendrite is slightly but not significantly different between the untreated condition and Forskolin condition (Tukey HSD *post hoc* test, *p* = 0.9) and between the untreated condition and H-89 condition (Tukey HSD *post hoc* test, *p* = 0.9). Also on the distal dendrites treatment with KN93 significantly reduces the expression of Cx36 compared to control conditions (Tukey HSD *post hoc* test, *p* < 0.01). On average the distal dendrites expressed 1.3 ± 0.5 Cx36 puncta/100 μm in KN93 treated slices (*n* = 7, Figure 2F). These results show that activation of PKA down-regulates tracer coupling not by removing the Cx36 GJs from the membrane and therefore it is most likely that the activation PKA reduces the opening probability of GJs. The inhibition of CaMKII activity does reduces the number of Cx36 puncta on the proximal and distal dendrites of olivary neurons and therefore activation of CaMKII is involved in the incorporation of Cx36 GJs at the site of electrical synapse. Despite the fact that activation of PKA and inhibition of CaMKII both reduce the number of tracer-coupled olivary neurons, the current results indicate that they use a different mechanism.

### Cx36 Gap Junctions in CaMKII Mutants

If CaMKII is required for the incorporation of Cx36 GJs at the site of the electrical synapse, as indicated by our pharmacological study, then genetic removal of the CaMKII protein should have a detrimental effect on the formation of the electrical synapse. Therefore, we studied the amount of GJs in αCaMKII and βCaMKII knock-out mice at an immunohistochemical as well as at the ultrastructural level. For the immunohistochemical analysis, volumetric samples (10.6 10^3^ μm^3^) were taken from IOs of αCaMKII−/−, βCaMKII−/− mutants and wild type littermates (control). Using the immunohistochemical approach a density of 28.8 ± 5.4 Cx36 puncta/1000 μm^3^ was found in the WT mice (*n* = 6), whereas lower densities were found in the αCaMKII−/− mutant mice (23.8 ± 3.8 Cx36 puncta/1000 μm^3^, *n* = 6) and in the βCaMKII−/− mutant mice (5.7 ± 2.0 Cx36 puncta/1000 μm^3^, *n* = 9; one-way ANOVA, *F*_(2,18)_ = 13.7, *p* < 0.01; Figure 3E). The density of Cx36 puncta in the IOs of βCaMKII−/− mutant mice were significantly smaller than in WT mice (Tukey HSD *post hoc* test, *p* < 0.01) and in αCaMKII−/− mutant mice (Tukey HSD *post hoc* test, *p* < 0.01; Figure 3E).

Ultrastructural analyses of GJs in WT mice show characteristics consistent with earlier studies (De Zeeuw et al., 2003; Van Der Giessen et al., 2006). WT GJs are present in the IO with a density of 87 ± 4 GJs/mm^2^ (*n* = 5, Figure 3F1), consist of a plaque with an average diameter of 312 ± 24 nm (*n* = 18, Figure 3F2) and an interneuronal space of 3.8 ± 0.2 nm thickness (*n* = 18, Figure 3F3); they show electron-dense deposits at both sides of the membrane and have attachment plaques surrounding the plaque with gap-junction channels (Figures 3A,B). For both the GJ density and interneuronal space, a significant interaction was found between the three CaMKII groups (GJ density: one-way ANOVA, *F*_(2,12)_ = 282, *p* < 0.01; GJ interneuronal space: one-way ANOVA, *F*_(2,26)_ = 8.1, *p* < 0.05). No significant interaction was found between the three groups and the diameter of GJs (one-way ANOVA, *F*_(2,26)_ = 2.2, *p* = 0.13). In αCaMKII−/− mutant mice, we observed GJs with normal characteristics as mentioned previously. The density of GJs in αCaMKII−/− mutant mice, 96 ± 9 GJs/mm^2^ (*n* = 4), was not significantly different from WT mice (Tukey HSD *post hoc* test, *p* = 0.25). Whereas in βCaMKII−/− mutant mice, the density of GJs in the olivary subnuclei was significantly smaller than in WT and αCaMKII−/− mice (1 ± 1 GJs/mm^2^, *n* = 6; Tukey HSD *post hoc* test, *p* < 0.01). Despite the lack of GJs in βCaMKII−/− mutant mice, the morphological characteristics of all glomeruli were comparable to WT (Figure 3C1). The vast majority of glomeruli exhibited two dendritic spines in the core of glomeruli surrounded by several excitatory and inhibitory terminals nonetheless centrally located GJs were missing. Moreover, the extraglomerular neuropil including dendritic lamellar bodies and glial GJs appeared normal. In the entire area of βCaMKII−/− IO that we scanned, we found only one GJ which had a normal interneuronal space (3.9 nm) compared to WT, however with a smaller plaque diameter (135 nm; Figure 3F2). We also observed one adhesion-plaque structure, which was not observed in previous studies (Figure 3C2). The GJ interneuronal space was significantly wider in αCaMKII−/− mutant mice (5.9 ± 0.6 nm, *n* = 10; Tukey HSD *post hoc* test, *p* < 0.01; Figure 3D) than in WT (3.8 ± 0.2 nm, *n* = 18) and in βCaMKII−/− mutant mice (3.9 nm, *n* = 1).

We conclude from these data that absence of βCaMKII leads to a strong reduction of GJs in all subnuclei of the IO. Despite the lack of βCaMKII, general ultrastructural morphology of olivary glomeruli is unchanged.

## Discussion

Electrical synapses are present in many areas of the brain and they have important roles in regulating the communication between clusters of neurons. In the IO, GJs are formed by Cx36 (Placantonakis et al., 2004; Van Der Giessen et al., 2006) and their presence is essential to support and synchronize physiological oscillations in the network (De Zeeuw et al., 1996, 2003; Long et al., 2002), which govern time-dependent motor learning (Van Der Giessen et al., 2008). So far, the relevance of electrotonic coupling in the IO has been explored by removal of GJs via genetic manipulations or by blocking the GJs with pharmacological agents (Long et al., 2002; Placantonakis et al., 2004, 2006; Leznik and Llinás, 2005; Martin and Handforth, 2006; Urbano et al., 2007; Van Der Giessen et al., 2008). In the present work, we demonstrate how two protein kinase pathways can modulate the electrotonic coupling between olivary neurons at a structural level. The importance of PKA and CaMKII was investigated using the tracer transfer method (Abbaci et al., 2008), which provides a measurable index of the extent of the coupled network. For all the experiments, we relied exclusively on passive diffusion of the tracer, because recently it has been shown, with thalamic Cx36 GJs, that repetitive neuronal activity (in order to actively load the cell with tracer) leads to a long-term depression of the conductance of GJs (Haas et al., 2011). Compared to an iontophoresis study in rat (Hoge et al., 2011), our results in mice showed a higher number of coupled neurons (15.3 ± 3.2 vs. 10.2 ± 3.3 cells), which might be explained by the different animal species or by the activity-induced depression of Cx36-GJs (Haas et al., 2011).

Our results demonstrate a Forskolin-mediated reduction in tracer-coupling between olivary neurons. Forskolin activates adenylyl cyclase, which up-regulates intracellular cAMP and the latter subsequently activates PKA. The reduction of tracer-coupling, we observed, is likely caused by indirect activation of PKA and is in line with the results obtained in the retina by Urschel et al. (2006) and Kothmann et al. (2009). However, it is still unclear to what extent the phosphorylation state of Cx36 reduces the coupling. Urschel et al. (2006) claim that PKA directly phosphorylates Cx36 and thereby affects the coupling, whereas Kothmann et al. (2009) conclude that activation of PKA triggers a protein-phosphatase2A-mediated dephosphorylation of Cx36 GJs. In our work, we could not check for the phosphorylation state of Cx36 GJs in the IO, but we could confirm that an increased PKA activity down-regulates the tracer-coupling between olivary neurons. The block of PKA with H-89, however, exerted a non-significant reduction in the amount of coupled neurons, which can best be explained by low basal PKA activity in the IO (under our experimental conditions).

In our experiments, enhancing PKA activity or inactivating CaMKII had the same effect on the tracer-coupling between olivary neurons, but the mechanisms underlying this effect is probably different. The number of dendritic Cx36 puncta counted in KN93 condition was smaller than the one in control condition, whereas the Forskolin and H-89 treatments did not induce any significant change in the number of dendritic Cx36 puncta. PKA predominantly acts on Cx36 phosphorylation sites S110 and S293 (Urschel et al., 2006; Kothmann et al., 2009), whereas CaMKII has its preferential sites of action on S110, T111 and S315 (Alev et al., 2008). Thus, the two kinases only partially share action sites on Cx36 and this may explain the different structural effects observed size and number of GJs. Our results showed that PKA-mediated down-regulation of tracer-coupling does not correlate with a reduction in the number of Cx36 puncta. The most plausible explanation is that increased activity of PKA reduces the open probability of the Cx36, thereby reducing the GJ conductance. In both cases, activation of PKA and inhibition of CaMKII, the down-regulation of the tracer-coupled network should be accompanied by an increase in input resistance. However, the changes in membrane parameters were not statistically significant. The extensive dendritic arborization of IO neurons and distal localization of electrical synapses preclude the possibility to assess GJ modulation with single patch clamp recordings (Wilders and Jongsma, 1992; Prinz and Fromherz, 2003).

Turecek et al. (2014) blocked NMDAR strengthening of electrotonic coupling between two IO neurons with KN-93, and they suggested that KN-93 does not affect baseline coupling, which is not fully in line with our findings. This difference in results might reflect the fact that Turecek et al. (2014) explored IO coupling after minutes of drug application (as opposed to hours as in our case) and at lower concentrations (1 vs. 10 μM).

NMDA receptor-dependent excitatory inputs on olivary neurons causes the necessary increase of intracellular Ca^2+^ to activate CaMKII (Turecek et al., 2014). Blocking the activation of CaMKII leads to a reduction in coupling, which is consistent with previous findings in the retina (Pereda et al., 1998; Kothmann et al., 2012). Furthermore, our results show, both at the immunohistochemical and ultrastructural level (Figures 3E,F1), that βCaMKII (but not αCaMKII) is necessary for Cx36 expression and electrical synapse formation in the IO. The mechanism by which βCaMKII can regulate the number and size of electrical synapses may involve a modification of the turnover of GJs. βCamKII possesses an actin–binding domain and has been implicated in actin stabilization/bundling at the synapse (Okamoto et al., 2009). Actin dynamics can regulate protein synthesis and protein degradation processes at the synapse. Connexin proteins have a short half-life time of 1–3 h (Laird, 1996) and influencing the Cx36 synthesis, insertion and degradation process may result in an alteration of the GJ coupling strength between neurons in a short time span (Musil et al., 2000; Djakovic et al., 2009).

In short, the sensitivity of Cx36 GJs to protein kinases like PKA and CaMKII is in line with the view that also electrical synapses can undergo long-term modification of their conductance (Landisman and Connors, 2005; Haas et al., 2011). In the present work, we show for the first time the different mechanisms of action of these pathways on the coupling of IO neurons. The modulatory effects of the two mediators examined in the present work might be fundamental for optimal performance and learning of movements (Lang, 1995; Van Der Giessen et al., 2008).

## Author Contributions

PB, SCI and MTGJ conceived the study. PB, SCI, MTGJ, CIDZ and YE designed the study. PB, SCI, EDH and RSG conducted the experiments and analyzed all data. YE provided CaMKII mutants. PB and MTGJ wrote the manuscript. All authors edited the manuscript and approved the final version.

## Conflict of Interest Statement

The authors declare that the research was conducted in the absence of any commercial or financial relationships that could be construed as a potential conflict of interest.
